# The Role of Organizational Factors and Human Resources in the Provision of Dental Prosthesis in Primary Dental Care in Brazil

**DOI:** 10.3390/ijerph17051646

**Published:** 2020-03-03

**Authors:** Maria Aparecida Cunha, Mario Vianna Vettore, Thiago Resende dos Santos, Antônio Thomaz Matta-Machado, Simone Dutra Lucas, Mauro Henrique Nogueira Guimarães Abreu

**Affiliations:** 1 Department of Community and Preventive Dentistry, Universidade Federal de Minas Gerais, Belo Horizonte 31270.900, Brazil; saudebucalbruma@gmail.com (M.A.C.); mariovettore@gmail.com (M.V.V.); simonedlucas@gmail.com (S.D.L.); 2 Department of Statistics, Universidade Federal de Minas Gerais, Belo Horizonte 31270.900, Brazil; thiagors007@gmail.com; 3 Department of Preventive and Community Medicine, Universidade Federal de Minas Gerais, Belo Horizonte 30130.100, Brazil; thomaz@nescon.medicina.ufmg.br

**Keywords:** public health, primary health care, dental prosthesis

## Abstract

This study aimed to investigate factors associated with dental prosthesis procedures by oral health teams (OHTs) in the Brazilian primary health care in 2013–2014, who participated in the National Program for Improving Access and Quality of Primary Health Care (PMAQ-AB). This is an analytical cross-sectional study using a questionnaire with dichotomous questions applied in 18,114 OHTs. The dependent variable studied was making any type of prosthesis (removable or fixed). Independent variables involved issues related to human resources and health service management. Data were submitted to simple and multiple binary logistic regression with odds ratio calculation, 95% confidence intervals, and p-values. Most OHTs (57%) do not perform any dental prosthesis. The teams that are more likely to perform dental prostheses have human resources-related characteristics, such as professionals admitted through public examinations (OR 1.25, 95% CI 1.14–1.36) and those involved in permanent education (OR 1.13, 95% CI 1.02–1.26). Moreover, OHTs with a more organized work process and that receive more significant support from municipal management are more likely to perform dental prostheses (p < 0.05). The oral health teams which tended to provide the most dental prostheses to benefit patients were; hired as civil servants, had a municipal career plan, involved all members of the oral health team, and trained undergraduate dental students from outreach programs. Better organizational support and improved work incentives may be needed to get the majority of oral health teams to start providing dental prostheses to their patients.

## 1. Introduction

Tooth loss is still relevant in public health despite the considerable global decline over recent years [1,2]. Brazilian older adults aged 65–74 years have a high prevalence of tooth loss. According to the 2010 Brazilian Oral Health Survey, 53.7% of this age group was edentulous [3]. Also, 63.1% and 37.5% of older adults in Brazil have upper and lower dentures, respectively. The need for at least one denture was 33.3% [4]. Tooth loss may affect psychological and social well-being, whereas prosthetic rehabilitation can restore oral health functions (e.g., chewing) and esthetics, improving oral health-related quality of life, minimizing the consequences of edentulism [5].

Prosthetic dental treatment is a relevant issue in dental health services planning and use, and a challenging component of health care systems in most countries due to several reasons [6]. The population aging is a demographic phenomenon occurring in Brazil and in other countries, which increases prosthetic dental treatment needs. Tooth loss is no longer considered a natural phenomenon among older adults, and the demand for tooth replacement in this age group has increased over the recent decades. Prosthetic treatment is highly costly for public dental services as it involves providing dental rehabilitation procedures services and laboratory expenses [3,4,5,6,7]. Access to dental prosthesis treatment is usually hampered by the low availability of this procedure within public health care and due to the limited socioeconomic status of the population [8]. 

In Brazil, dental prosthetic procedures were exclusively offered in secondary dental care units supported by the regional dental prosthesis laboratories [9]. However, since 2004, the National Oral Health Policy supports the provision of total and partial removable and fixed dental prosthesis within primary health care (PHC) services [10], taking into account the comprehensiveness of care and the high demand for dental prostheses among adults and the elderly population in Brazil. Thus, it is paramount to understand how dental prosthesis procedures are being performed in PHC within the Brazilian public health system.

In 2011, the Brazilian Ministry of Health developed a proposal to evaluate the quality of PHC services at the national level, namely the National Program for Improving Access and Quality of Primary Health Care (PMAQ-AB). Ultimately, the results from PMAQ-AB would provide information for financial onlendings to local health care teams as an incentive according to their performance over time. To date, two rounds of PMAQ-AB have been developed, between 2011 and 2012, and between 2013 and 2014. Previous studies based on the PMAQ-AB database have evaluated the performance of dental clinical procedures in PHC [11,12], preventive oral health [13] procedures, as well as the relationship between PHC and specialized care [14]. A recent study reported an unequal geographical distribution of the performance of dental procedures in PHC in Brazil [15]. Although this topic has been rarely explored, previous evidence suggests that the work organization of health care teams, planning activities, and human resources factors are related to the performance of oral health teams in PHC [16,17,18]. The management of the public health care system, including the human resources in health care, must be structured in order to meet the demands and challenges of the population [19,20]. Therefore, better structured oral health care teams in PHC may supposedly show better outcomes in the health care system.

Recent studies have adopted the Andersen and Davidson behavioral model of health services use to examine the predictors of dental services use [21,22,23,24]. This model bundles the determinants of health services use into individual and contextual predisposing, enabling and need characteristics [25]. Contextual factors are the environment and circumstances of health care that facilitates the access and use of health services. Enabling contextual factors involve local and national health policies, financing, and organization of health care [21,22]. Organizational factors related to health services use may encompass the amount and distribution of health services facilities and personnel, including human resources and working processes, as well as how they are structured to provide services. Previous research assessing enabling contextual factors on dental services use focused on the association of expenditure in health care and population coverage by primary care with dental services use [21,22]. Nonetheless, evidence of the influence of the organization of health care system on the use of oral health services is scarce.

An improved understanding the role of organizational factors in the provision of dental prostheses within the Brazilian primary health care system could generate information to improve the work process of oral health professionals. Also, enhancing the organization of primary dental care services could help increase patient access to specialized dental services.

This study investigated the association between organization factors, including human resources and work process characteristics, and provision of the dental prosthesis by the oral health teams in a nationally representative sample of Brazilian dentists working in primary health care using the National Program for Improving Access and Quality of Primary Health Care (PMAQ-AB) 2013–2014.

## 2. Materials and Methods

### 2.1. Study Design and Participants

This was a cross-sectional analytical study in which data from the second round of National Program for Improving Access and Quality of Primary Health Care (PMAQ-AB) in the primary health care teams that was conducted by the Ministry of Health in Brazil in the 2013–2014 period. The PMAQ-AB evaluation builds on the Donabedian model that establishes a fundamental framework for examining health services and evaluating the quality of health care based on the concepts of structure, process, and result [11]. PHC principles, including integrality and coordination, are also considered in the PMAQ-AB evaluation [26].

The participants consisted of dentists who worked in the dental teams in primary health care, and were selected for the second round of PMAQ-AB. Each oral health team consists of one dentist, one dental assistant, and, in some teams, one oral health technician. In total, 23,251 oral health teams were deployed in Brazil in the initial phase of the PMAQ-AB. In the first round of PMAQ-AB, 50% of oral health teams could participate in the program, while in the second round, municipal administrators appointed the oral health teams they deemed fit to participate. This resulted in a total of 18,114 (77.9%) oral health teams in the second round of PMAQ-AB.

### 2.2. Data Collection

Information concerning human resources and work process characteristics were obtained through interviews with dentists and documents related to the PHC unit. Forty-six Brazilian institutions in higher education and research institutes were involved in data collection, including the interviews and document retrieval. The interviews were conducted by 989 trained health professionals using a questionnaire with structured questions specifically developed for the research. All interviewers participated in a 40-hour training involving primary health care content, research methods, and PMAQ questionnaires. A formal evaluation assessed interviewers’ skills to collect data using a standardized approach. A supervisor was appointed for every three interviewers. 

The interviews were carried out using an electronic questionnaire operationalized through a mobile app. The completed questionnaires were sent via the Internet to a central database hosted at the Ministry of Health that was also responsible for consistency analysis and the certification of the primary health care teams. 

### 2.3. Variables

The outcome of the study was the provision of removable or fixed dental prosthesis by the oral health teams, built according to the following question: “does the oral health team provide removable complete dental prostheses” (yes/no); “does the oral health team provide removable partial denture” (yes/no); “does the oral health team provide fixed prosthesis” (yes/no); “does the oral health team provide temporary prosthesis” (yes/no). 

Organization factors included human resources and work process characteristics related to the dental care system [27]. The former included the following variables: seniority of the professional in the current oral health team (up to 2 years, more than 2 years); type of employer (direct administration, others); type of working contract (civil servant, others); admission process (public tender, others); municipal career development plan (yes, no, information not available); participation in seminars, workshops and group discussions within the PHC unit (yes, no); professionals of the oral health team involved in Continuum Professional Development (CPD) (All members of the oral health team, dentist or any dental assistant, information not available); CPD sessions consider the perceived needs of the oral health team (yes, no, information not available); oral health team hosts undergraduate dental students from outreach program (yes, no, information not available); higher degree (Specialization, Master of Science or Ph.D., undergraduate degree).

Work process variables [27,28] included assessment of dental prosthesis needs of the population (yes, no); planning and scheduling activities of the oral health team on a monthly basis (yes, no); monitoring and analysis of oral health indicators (yes, no); receives financial support for planning and organization of the work process (yes, no); receives management support from the municipal health department (yes, no); attends the primary healthcare team meetings (always/sometimes, never); goals in primary health care agreed with the municipal health department (yes, no); existence of specialized dental services (Dental Specialty Centers) (yes, no). 

### 2.4. Data Analysis

A descriptive analysis was used to report the frequency and proportion of human resources and work process variables. Odds ratios (ORs), 95% confidence intervals (95% CI) and p-values were estimated through binary logistic regression models to test the association between human resources, work process variables and provision of the dental prosthesis by the oral health teams. All variables with a p-value < 0.25 in the bivariate analysis were included in the multiple logistic regression model using the Forward Wald method. Variables with p-value < 0.05 were retained in the final model. Multicollinearity tests were performed among the independent variables through the evaluation of variance inflation factors (VIF).

The Hosmer–Lemeshow (HL) test was used to evaluate the quality of adjustment of logistic regression models in this study, with the correction proposed by Paul et al. [28] (2013) applied to large samples. The correction mainly consists of changing their number of groups (*m*) by sample size [28]. Also, a residual analysis was performed to evaluate the suitability of the binary logistic regression model adjusted to the data. All statistical analyses were performed using SPSS for Windows version 19.0 (IBM, Chicago, IL, USA).

### 2.5. Ethical Aspects

The present study was approved by the National Ethics Research Council and by the Research Ethics Committee of the Federal University of Minas Gerais (CAAE 02396512.8.0000.5149). The participation of the dentists in the interviews was voluntary, and they could withdraw from the study at any time.

## 3. Results

Of the 18,114 oral health teams evaluated, most (57%) did not provide any type of dental prosthesis.

Most oral health teams consisted of professionals working up to two years in the current oral health team. Most oral health professionals were hired by the direct administration in non-statutory employment status. Half of the participants were admitted through public examination, and around 63% do not have a career plan in their current job. Most oral health professionals (63.5%) participated in seminars, workshops, and group discussions. Most of the professionals of the oral health teams were involved in CPD, and only 12.7% of the oral health teams hosted undergraduate dental students from outreach programs, and nearly 30% had a specialization, Master of Science or Ph.D. Assessment of dental prosthesis needs of the population was conducted by 52.5% of oral health teams. Most oral health teams plan and schedule activities monthly, monitor and analyze oral health indicators, and receive financial support for planning and organization of the work process, information from the health surveillance system, and local epidemiological information. Most of the oral health teams attend primary health care team meetings and agree on the goals in primary health care with the municipal health department (Table 1).

The multivariable logistic regression model was adjusted to the data using the corrected HL test, and the model was adequately adjusted (p-value > 0.05). The normal probability plot of the residuals (probability envelope) also indicated the adequacy of the model. Based on this adjusted model, the point and interval estimates and the p-values for the OR associated with the independent variables are shown in Table 1.

All independent variables related to human resources and work processes were statistically associated with the provision of dental prostheses procedures at Primary Dental Care in Brazil in the crude analysis. In the adjusted logistic regression analysis, oral health teams where the professional was hired as a civil servant (OR: 1.25, 95% CI 1.14–1.36) were more likely to provide dental prosthesis. The likelihood of providing dental prostheses were significantly lower for oral health teams where there was no municipal career plan (OR: 0.85, 95% CI 0.76–0.94). Oral health teams where all members of the oral health team were involved in CPD (OR: 1.13, 95% CI 1.02–1.26) and those hosting undergraduate dental students from outreach programs (OR: 1.42, 95% CI 1.27–1.59) were more likely to provide dental prosthesis. Lack of assessment of dental prosthesis needs at population level (OR: 0.24, 95% CI 0.22–0.26), lack of planning and scheduling activities on a monthly basis (OR: 0.86, 95% CI 0.77–0.97), and lack of monitoring and analysis of oral health indicators (OR: 0.88, 95% CI 0.80–0.96) decreased the odds of provision of dental prosthesis. Not receiving management support from the municipal health department (OR: 0.24, 95% CI 0.22–0.26) and absence of specialized dental services (OR: 0.44, 95% CI 0.40–0.48) also reduced the likelihood of providing dental prosthesis.

## 4. Discussion

This is the first investigation of the role of organizational factors in the provision of dental prostheses within the Brazilian primary health care system using the PMAQ-AB survey. Our findings support the role of different characteristics related to human resources and the work process of dental teams working in primary health care in the provision of dental prosthesis in Brazil. The provision of dental prosthesis in primary dental care was positively associated with specific characteristics of the oral health teams such as admission on the health care system through public examination and involvement in CPDs. The human resources characteristics of the municipality were also important factors related to the provision of dental prosthesis, including having a career plan, management support from the municipal health department, and the existence of specialized dental services. The use of epidemiological information, such as assessment of dental prostheses’ needs of the population, and monitoring and analysis of oral health indicators were also meaningful aspects associated with the provision of dental prosthesis in primary dental care.

Prosthetic rehabilitation in primary health care has been recently incorporated into the Brazilian National Health Service. Therefore, we expected that dental prostheses in primary care would not be provided by all oral health teams in Brazil. The reasons for such findings are linked to professional issues (e.g., insecurity in performing prosthesis and lack of training), and structural problems such as lack of consumables and instruments, damaged equipment, and a large number of patients with clinical dental needs [17].

The association between the provision of dental prosthesis and human resources variables was identified. Previous evidence has shown that adequate human resource management is essential for the effectiveness of the health system and the provision of health care [17,18]. Involvement in CPDs by all members of the oral health team increased the likelihood of the provision of dental prosthesis. The updating and qualification of professionals through permanent education is a crucial point to work process quality. According to Tesser et al. (2011) [29], permanent education contributes to the construction of values, qualifying, and enriching health work management. All this improves the process of knowledge and analysis of social reality, increasing resolution, humanization, and coordination of care. Interestingly, the attendance of postgraduate courses and participation in seminars, workshops, and group discussions within the primary health care unit were not related to the provision of dental prosthesis. 

Oral health teams involved in oral epidemiological surveillance were more likely to provide dental prostheses. Understanding population oral health needs requires coordinated actions guided by epidemiology, planning, and management of the health services [30]. Hannaford (2005) [31] affirms that epidemiology is an excellent tool for understanding the health needs of the population assisted by primary health care. Thus, it is possible to provide data on the needs of the community, carrying out improved health care planning. In this regard, it is necessary to monitor epidemiological indicators and evaluate the proposed actions. Despite the lack of specific studies assessing the impact of these variables in the performance of dental prostheses, it seems that the association identified in this study has such mediations. Other general health surveillance actions seem not to be a factor associated with the performance of dental prosthesis.

Hosting undergraduate dental students from outreach programs in PHC increases the likelihood of providing dental prostheses. It can be argued that the production of knowledge allows transforming health services, focusing on the quality of care. The exchange of knowledge between teaching-service personnel facilitates the support to health teams and carrying out new strategies and actions. The work process organization suggests that oral health teams require support for community-level initiatives [25] to address oral health needs.

Oral health teams that plan their activities routinely and those who receive financial support for planning and organization of the work process from the central level are more likely to provide dental prosthetic procedures because they are more organized and engaged. Previous studies have shown that organizational planning within the health system is critical because it embraces skills development, improvement of organizational structures, allocation of resources, and the development of partnerships and health policies [19,32]. Thus, a clear understanding of the local context results in better conditions to provide health care. The amount of support received by the primary health care teams to perform organizational activities in the work process was associated with the improved quality and management of the health teams [19].

The organization of the healthcare system can enhance the integrality of care at various levels, which is also a relevant factor for the improvement of the oral health team. Lino et al. (2014) [20] state that the mere existence of different health care levels is insufficient to improve health care, and the interrelationship between these levels is paramount for the effectiveness of the health care system. In Brazil, specialized dental services correspond to secondary oral healthcare, which is the reference for primary dental care, and are responsible for offering specialized, complex, and complementary procedures to primary health care [14]. No specific studies have been conducted yet to examine the association between the provision of dental prostheses in primary health care and the presence of specialized dental services. However, oral health teams within primary care that can refer a complex case to secondary oral health care reflect a more articulated and organized healthcare network [22], which explains the higher likelihood of these teams providing dental prostheses. Also, the fact that dental prostheses have traditionally been offered in secondary care in Brazil may influence their performance in primary health care in municipalities that offer secondary care.

The second cycle of PMAQ-AB relied on the adherence of oral health teams selected by municipal managers according to their availability to participate in the evaluation. This may have resulted in selection bias since the most structured teams were selected. Information bias might have occurred in this study since the data was collected in a questionnaire completed by the professionals from the oral health teams. The lack of data on dental prostheses’ needs and demographic variables may also be a limitation of this study. However, a previous publication has shown that the dental prostheses’ needs in Brazil did not seem to be a relevant predictor to explain their performance. For example, the North region showed a high rate of dental prostheses’ needs, but this region has one of the lowest provision of dental prostheses [15]. Moreover, a multilevel approach would be necessary for a better understanding of contextual determinants of dental prostheses procedures. The findings of this study show the relevance of human resources management and the importance of aspects related to the support of municipal management for the organization of the work process and ensuing improvement of comprehensive health care, such as dental prostheses provision. 

## 5. Conclusions

The oral health teams which tended to provide the most dental prostheses to benefit patients were; hired as civil servants, had a municipal career plan, involved all members of the oral health team, and trained undergraduate dental students from outreach programs. Better organizational support and improved work incentives may be needed to get the majority of oral health teams to start providing dental prostheses to their patients.

## Figures and Tables

**Table 1:** Association of human resources and work process variables with provision of dental prostheses in Primary Dental Care in Brazil.

Variable	N (%)	Crude OR (95% CI)	P-Value	Adjusted OR (95% CI)	P-Value
*Human Resources*					
Seniority of the professional in the current oral health team					
Up to 2 years	10,373 (57.3%)	1			
More than 2 years	7741 (42.7%)	1.21 (1.14–1.28)	<0.001		
Type of employer					
Direct Administration	14,871 (82.1%)	1			
Others	3243 (17.9%)	1.11 (1.03–1.20)	<0.001		
Type of working contract					
Others	10,121 (55.9%)	1			
Civil servant	7,993 (44.1%)	1.19 (1.12–1.27)	<0.001		
Admission process					
Others	9065 (50.0%)	1		1	
Public tender	9049 (50.0%)	1.32 (1.25–1.41)	<0.001	1.25 (1.14–1.36)	<0.001
Municipal career development plan *					
Yes	2986 (16.5%)	1		1	
No	11,468 (63.3%)	0.69 (0.64–0.75)	<0.001	0.85 (0.76–0.94)	<0.001
Participation in seminars, workshops and group discussions within the PHC unit					
Yes	11,504 (63.5%)	1			
No	6610 (36.5%)	0.66 (0.62–0.70)	<0.001		
Professionals of the oral health team involved in CPD *					
Dentist or any dental assistant	2622 (14.5%)	1		1	
All members of the oral health team	12,090 (66.7%)	1.38 (1.25–1.51)	<0.001	1.13 (1.02–1.26)	<0.001
CPD sessions consider the perceived needs of the oral health team *					
Yes	10,959 (60.5%)	1			
No	3753 (20.7%)	0.81 (0.76–0.88)	<0.001		
Oral health team hosts undergraduate dental students from outreach program *					
No	2296 (12.7%)	1		1	
Yes	12,416 (68.5%)	1.68 (1.54–1.84)	<0.001	1.42 (1.27–1.59)	<0.001
Higher degree					
Specialization, Master of Science or PhD	5533 (30.5%)	1			
Undergraduate degree	12,581 (69.5%)	1.18 (1.10–1.25)	<0.001		
*Work process*					
Assessment of dental prosthesis needs of the population					
Yes	9504 (52.5%)	1		1	
No	8610 (47.5%)	0.20 (0.19–0.21)	<0.001	0.24 (0.22–0.26)	<0.001
Planning and scheduling activities of the oral health team on monthly basis					
Yes	14,950 (82.5%)	1		1	
No	3164 (17.5%)	0.87 (0.79–0.96)	<0.05	0.86 (0.77–0.97)	<0.05
Monitoring and analysis of oral health indicators					
Yes	12,031 (66.4%)	1		1	
No	6083 (33.6%)	0.58 (0.55–0.63)	<0.05	0.88 (0.80–0.96)	<0.05
Receives financial support for planning and organization of the work process					
Yes	14,303 (79.0%)	1			
No	3811 (21.0%)	0.58 (0.54–0.62)	<0.001		
Receives management support from the municipal health department					
Yes	14,925 (82.4%)	1		1	
No	3,189 (17.6%)	0.20 (0.19–0.21)	<0.001	0.24 (0.22–0.26)	<0.001
Receives information from the health surveillance system					
Yes	14,454 (79.8%)	1			
No	3,660 (20.2%)	0.62 (0.57–0.66)	<0.001		
Receives local epidemiological information					
Yes	14,518 (80.1%)	1			
No	3596 (19.9%)	0.58 (0.54–0.62)	<0.001		
Attends primary health care team meetings					
Always/sometimes	16,918 (93.4%)	1			
Never	1196 (6.6%)	0.51 (0.45–0.58)	<0.001		
Goals in primary health care agreed with the municipal health department					
Yes	15,541 (85.8%)	1			
No	2573 (14.2%)	0.65 (0.59–0.70)	<0.001		
Existence of specialized dental services					
Yes	11,178 (61.7%)	1		1	
No	6936 (38.3%)	0.36 (0.34–0.39)	<0.001	0.44 (0.40–0.48)	<0.001

* For these variables there are some missing data. OR: Odds Ratio; CI: Confidence Interval; PHC: Primary Health Care; CPD: Continuum Professional Development.
